# Authors' Responses to Peer Review of “Influence of the COVID-19 Lockdown on the Physical and Psychosocial Well-being and Work Productivity of Remote Workers: Cross-sectional Correlational Study”

**DOI:** 10.2196/34609

**Published:** 2021-12-01

**Authors:** Yessica Abigail Tronco Hernández, Fabio Parente, Mark A Faghy, Clare M P Roscoe, Frances A Maratos

**Affiliations:** 1 School of Health Professions University of Plymouth Plymouth United Kingdom; 2 School of Psychology College of Health, Psychology and Social Care University of Derby Derby United Kingdom; 3 School of Human Sciences College of Science and Engineering University of Derby Derby United Kingdom

*This is the authors’ response to peer-review reports for the paper “Influence of the COVID-19 Lockdown on the Physical and Psychosocial Well-being and Work Productivity of Remote Workers: Cross-sectional Correlational Study”.*

## Round 1 Review

### Anonymous

We are thankful to the reviewer [1] for the helpful suggestions regarding background literature. The first study is now mentioned in the first paragraph of the *Introduction* [2], in the context of income loss as a result of lockdown restrictions. The second study is now discussed in the first paragraph of the *Implications* section, in the context of a return to office-based work and contingencies that employers can implement to improve the psychophysical well-being of their workers.

### Reviewer AB

1. We are grateful to the reviewer [3] for the positive review of our manuscript.
2. We agree! Indeed, there is some scope within our project to track down a few of our respondents to assess changes in their psychophysical well-being and work productivity since their first survey response, particularly now that the United Kingdom appears set to begin lifting most lockdown restrictions and to rescind the recommendation that people work from home where possible. We will consider the possibility and produce a follow-up study should this prove feasible (eg, sufficient sample size).

### Further Amendments

In addition to addressing the reviewers’ comments, we have taken heed of the editorial recommendations and implemented the following changes:

- Ensured the structured abstract has the recommended section headings.
- Ensured the major headings follow the IMRD schema.
- Ensured the major headings and subheadings follow the Microsoft Word style guidelines.
- Amended Table 3 as per the caption to ensure a good portrait fit.
- Changed all in-text citations from a superscript format to an [n] format.

We believe these changes have resulted in an improved revised manuscript, and we hope you will find they have been addressed satisfactorily.

We look forward to receiving an outcome regarding the publication of our manuscript in JMIRx Med.
